# A favorable inductive remission rate for decitabine combined with chemotherapy as a first course in <60‐year‐old acute myeloid leukemia patients with myelodysplasia syndrome features

**DOI:** 10.1002/cam4.2418

**Published:** 2019-07-19

**Authors:** Fengqi Liu, Hehua Wang, Junru Liu, Zhenhai Zhou, Dong Zheng, Beihui Huang, Chang Su, Waiyi Zou, Duorong Xu, Xiuzhen Tong, Juan Li

**Affiliations:** ^1^ Department of Hematology, The First Affiliated Hospital Sun Yat‐sen University Guangzhou China

**Keywords:** acute myeloid leukemia, chemotherapy, complete remission, decitabine, induction therapy, MDS features

## Abstract

In acute myeloid leukemia (AML), myelodysplasia‐related changes contribute to a poor prognosis. This retrospective, propensity score‐matched study analyzed 108 newly diagnosed AML patients with features of myelodysplasia syndrome (MDS) (aged 14‐60 years) from 2014 to 2018, who received either idarubicin and cytarabine (IA) or decitabine, idarubicin and cytarabine (DAC+IA), and compared efficacy and toxicity between the two regimens. After propensity score matching, there were 54 patients in each group. The rate of complete remission (CR) was higher in the DAC+IA group than in the IA group (85.2% vs 68.5%, *P* = .040) after the first course, and toxicities were comparable in both groups. Multivariate analysis indicated that the combination with DAC was independent factor for CR rate after the first induction therapy (OR = 2.978, 95% CI:1.090‐8.137, *P* = .033). Subgroup analysis showed a CR advantage for DAC+IA (vs IA) for patients of intermediate‐high risk status according to National Comprehensive Cancer Network prognostic stratification. In conclusion, DAC+IA is therefore offered as a new induction choice for newly diagnosed AML patients with features of MDS, aged <60 years old, especially in intermediate‐high risk status.

## 1 INTRODUCTION

Acute myeloid leukemia (AML) is a heterogeneous disease. IA, a regimen of 3 days of idarubicin (IDA) and 7 days of cytarabine (Ara‐C), has been one of the standard 3+7 induction treatments for AML. Generally, 30% to 40% of adult patients could not achieve satisfying outcomes.^1^ One of the factors that contributes to its poor prognosis, AML with myelodysplasia‐related changes (AML‐MRC),^2^, ^3^ includes a history of myelodysplasia syndrome (MDS), MDS‐related cytogenetic abnormalities, and multilineage dysplasia,^4^ with two of these conditions not involving an MDS history and accounting for over 30% of AML cases.^5^ Besides, part of AML patients also had some features of MDS such as a history of more than 6 months of macrocytic anemia and low percent of blasts in bone marrow, which were easy to be ignored. Considering the characteristic abnormalities of MDS in these patients, we assume that the addition of decitabine (DAC) to AML therapy may improve its induction effectiveness as DAC, a deoxyribonucleic acid (DNA) methyltransferase inhibitor (DNMTi), has already been approved for treating MDS^6^, ^7^ and elderly patients with AML.^8^ In this study, we retrospectively analyzed 108 newly diagnosed AML patients between 2014 and 2018, who were treated with either IA or DAC+IA as induction therapy, to compare the curative and side effects of DAC+IA to those of standard two‐drug induction therapy (IA).

### 1.1 Patients and methods

#### 1.1.1 Patients

In this study, we retrospectively analyzed newly diagnosed AML patients (excluding M3) in the department of hematology of the First Affiliated Hospital of Sun Yat‐sen University between January 2014 and December 2018 according to the 2008 World Health Organization (WHO) classification of myeloid neoplasms and acute leukemia.^9^ All patients were aged 14‐60 years; and therapy‐related leukemia and previously diagnosed blood disease, such as myelodysplastic/myeloproliferative neoplasm (MDS/MPN)‐transformed leukemia, were excluded. Moreover, the patients were required to meet at least one of the following standards: (a) Anemia, leucopenia, or thrombocytopenia for over 6 months; (b) Macrocytic anemia: mean corpuscular volume (MCV) >95.0 fL according to the reference value in our hospital^10^; (c) Observation of marrow dyshematopoiesis; (d) 20%‐30% immature cells in bone marrow; or (e) Chromosomal karyotyping or fluorescence in situ hybridization (FISH) detected −5/del(5q), −7/del(7q), i(17q)/t(17p), −13/del(13q), del(11q), del(12p)/t(12p), idic(X)(q13), +8, or del(20q).^4^ All consecutive patients who met the standards and received DAC+IA or IA regimen were included without selection. Patients underwent prognostic stratification and response estimation in accordance with the 2018 National Comprehensive Cancer Network (NCCN) guidelines.^11^ Finally, a total of 108 patients were enrolled.

### 1.2 Treatment protocols and outcomes

#### 1.2.1 Induction therapy

Patients in the DAC+IA group received DAC combined with an IA regimen as follows: Decitabine 20 mg/m^2^ intravenously for five consecutive days (days 1‐5), a standard dose of cytarabine (100‐200 mg/m^2^/d) intravenously for seven days (days 1‐7) with idarubicin (8‐10 mg/m^2^) intravenously daily for 3 days (days 1‐3). In the IA group, patients were treated with 8‐10 mg/m^2^ IDA (days 1‐3) combined with 100‐200 mg/m^2^/d cytarabine for days 1‐7.

Supportive care was given during treatment. Transfusions of blood products were also provided when necessary. Red blood cells (RBCs) were infused when hemoglobin was <60 g/L (6.0 g/dL) or symptoms of anemia were observed, while platelets were given to patients with platelets <20 × 10^9^/L (20 000 mcL) and those who showed any signs of bleeding. Subcutaneous granulocyte colony‐stimulating factor (G‐CSF) was injected when neutrophils were <0.5 × 10^9^/L (500 mcL) during the myelosuppression stage. Treatment‐related toxicities were evaluated with the Common Terminology Criteria for Adverse Events (CTCAE) version 3.0. Time to hematopoietic recovery was measured from the first day of the chemotherapy to the time when the neutrophil count was >0.5 × 10^9^/L (500 mcL)^12^ or the platelet count was higher than 20 × 10^9^/L (20 000 mcL). Only patients achieving complete remission (CR) were considered for the analysis of recovery.

### 1.3 Follow‐up treatment

**Figure 1:**
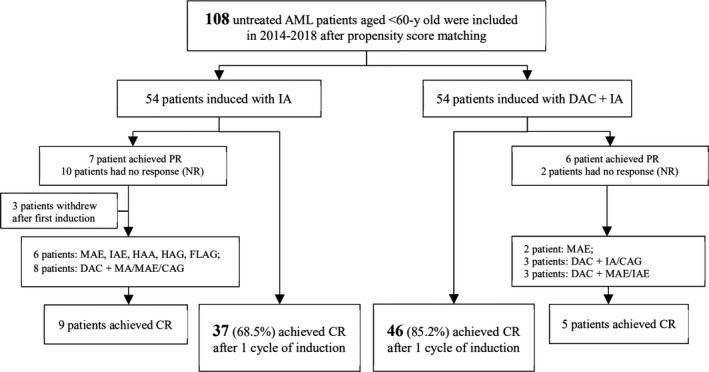
Enrollment and outcomes in patients with decitabine treatment. Abbreviations: AML, acute myeloid leukemia; CAG, Cytarabine + Aclarubicin +G‐CSF; CR, complete remission; DAC, Decitabine; FLAG, Fludarabine + Cytarabine +G‐CSF; HAA/HAG, Homoharringtonine + Cytarabine +Aclarubicin/ Granulocyte Colony‐Stimulating Factor (G‐CSF); IA, Idarubicin + Cytarabine; MAE/IAE, Mitoxantrone/ Idarubicin (MA) + Cytarabine + Etoposide); MDS, myelodysplasia syndrome; PR, partial remission

The treatment response was evaluated around 21‐28 days after chemotherapy and used to divide the patients into CR, partial remission (PR), and no response (NR), according to the 2018 NCCN clinical practice guideline of AML. Those who achieved CR after the first course of treatment entered consolidation treatment. The others received re‐induction (the applied regimens are shown in Figure 1), with consolidation treatment started if they achieved CR. Otherwise, patients who did not achieve CR after two cycles of induction therapy were regarded as induction failure and received salvage treatment. Relapse following CR was defined based on the reappearance of leukemic blasts in the peripheral blood or a finding of more than 5% blasts in bone marrow. Overall survival (OS) was measured from the date of diagnosis until death from any cause. Progression‐free survival (PFS) was calculated from treatment initiation to death or disease progression.

### 1.4 Statistical methods

We used propensity scoring to minimize bias and ensure similarities between the two treatment groups. Patients were matched with age, gender, initial white blood cell (WBC), history of symptoms, MCV, blasts in bone marrow, dysplasia, cytogenetics related to MDS, and NCCN prognostic stratification. By using a 1:1 nearest neighbor matching algorithm that pairs patients with the closest propensity scores within a defined limit (calipers of width equal to 0.20), the propensity score yielded two matched cohorts of 54 patients.

Chi‐square or Fisher's exact tests were used for the difference analysis of characteristics, CR rates, and the incidence of adverse events between two arms. The duration of cytopenia for different groups was compared by the Mann‐Whitney U test. For risk factor analysis of CR rates, the Logistic regression was used. The Kaplan‐Meier test was used to estimate OS and PFS. All reported *P* values are two‐sided, and *P* <.05 is considered statistically significant. All statistical analyses were performed using SPSS 19.0 software.

## 2 RESULTS

### 2.1 Baseline characteristics

Between January 2014 and December 2018, a total of 134 patients with a median age of 36 years old (range, 14‐60 years old) met the study inclusion criteria, with 60 and 74 treated with DAC+IA and IA regimen, respectively. Baseline characteristics for these patients (before propensity score matching) are listed in Table S1, and it did not vary significantly between the two groups.

To minimize the effects of treatment selection bias, adjustments were made using the propensity score matching method, and we identified 54 patients in each group. Patient characteristics used in the propensity score analysis are detailed in Table 1 and were well balanced between the two groups. The median age was 35.5 years (range, 14‐60) in the DAC+IA group and 35.0 years (range, 15‐59) in the IA group (*P* = .826). A bone marrow aspiration exam showed that dysplasia was found in 12 patients (11.1%). The results of karyotype analysis and FISH showed that 11 (10.2%) had at least one of the MDS‐related cytogenetic changes. Based on the 2018 NCCN criteria, 34 patients (31.5%) were classified as favorable‐risk, 37 (34.3%) as intermediate‐risk, and 37 (34.3%) as poor‐risk.

**Table 1:** Characteristics of 108 patients after propensity score matching

	IA (n = 54)	DAC+IA (n = 54)	*P* value
Age, years			
Median	35	35.5	.902
Range	15‐57	14‐59	
History, months			
Median	1.0	1.0	.841
Range	0.2‐7.0	0.1‐6.0	
WBC, ×10^9^/L			
Median	18.25	25.00	.808
Range	1.44‐361.0	1.27‐230.0	
MCV, fL			
Median	100.6	100.4	.837
Range	80‐115	69‐121	
Blasts, %			
Median	53.5	56.5	.888
Range	21.0‐94.0	21.0‐91.0	
Gender, n(%)			
Male	30 (55.6)	33 (61.1)	.696
Female	24 (44.4)	21 (38.9)	
FAB category, n(%)			
M0	1 (1.9)	2 (3.7)	.751
M1	6 (11.1)	5 (9.3)	
M2	22 (40.7)	19 (35.2)	
M4	6 (11.1)	6 (11.1)	
M5	19 (35.2)	20 (37.0)	
M6	0 (0.0)	2 (3.7)	
Dysplasia, n(%)			
0	48 (88.9)	48 (88.9)	1.000
1	6 (11.1)	6 (11.1)	
Cytogenetics related to MDS, n(%)			
0	49 (90.7)	48 (88.9)	1.000
1	5 (9.3)	6 (11.1)	
NCCN prognostic stratification, n(%)			
Favor	17 (31.5)	17 (31.5)	.784
Intermediate	17 (31.5)	20 (37)	
Poor	20 (37)	17 (31.5)	

Abbreviations: FAB, French‐American‐British classification; MCV, mean corpuscular volume; MDS, myelodysplasia syndrome; WBC, white blood cell.

### 2.2 Response to induction treatment

At the end of the first course of induction therapy, the DAC + IA group displayed a statistically significant increase in CR rates (85.2% vs 68.5%, *P* = .040; Figure 1). Among 22 patients without CR who were administered a second course of the induction, as shown in Figure 1, nine responded after chemotherapy combined with DAC and five after intense chemotherapy such as mitoxantrone/cytarabine/etoposide (MAE) and fludarabine/ cytarabine/G‐CSF (FLAG).

**Figure 2:**
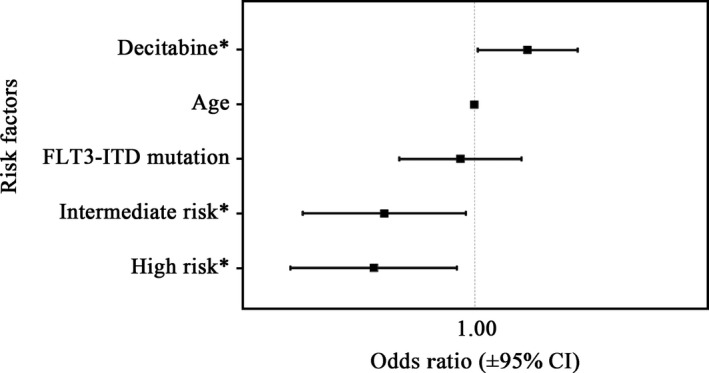
Multivariate analysis of complete remission rate. *P < .05

To further confirm the factors associated with the response of AML patients, we performed multivariate logistic regression analysis. Remarkably, CR rate was independently correlated with combinations including DAC (*P* = .033, OR 95% CI = 1.090 to 8.137) and NCCN risk status (Intermediate vs Favorable: *P* = .032, OR 95% CI: 0.032‐0.860; Poor vs Favorable: *P* = .019, OR 95% CI: 0.025‐0.715), which was adjusted for age and prognostic markers such as FLT3‐ITD (shown in Figure 2).

### 2.3 Subgroup analysis

**Figure 3:**
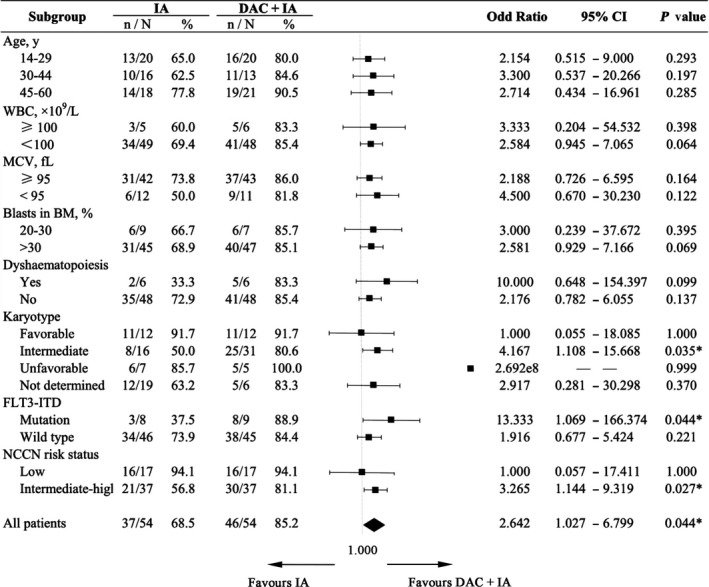
Subgroup analysis of complete remission rate after one course of induction. Abbreviations: MCV, mean corpuscular volume; BM, bone marrow. *P < .05

In an exploratory subgroup analysis using Logistic regression (Figure 3), a CR advantage for the DAC + IA compared with the IA could be demonstrated for patients with intermediate karyotype (*P* = .035) or FLT3‐ITD mutations (*P* = .044). Combining the cytogenetics with molecular results, a significant difference between DAC + IA and IA group was revealed in the subgroup of patients with intermediate‐poor risk status (56.8% vs 81.1%, *P* = .027).

### 2.4 Treatment‐related toxicity

In particular, the combinations including DAC did not add extra treatment‐related side effects, and toxicities were comparable in both groups.

To evaluate the hematologic toxicities listed in Table 2, we analyzed 83 patients who achieved CR after the first induction chemotherapy. All patients experienced WHO Grade 4 neutropenia and thrombocytopenia. The time to neutrophil recovery ranged from 8 to 36 days in DAC+IA group (median time, 20 days), whereas in the non‐DAC group, the time ranged from 16 to 24 days (median time, 19 days). The median time to platelet recovery for both DAC+IA and non‐DAC groups was 18 days. Neither the time to neutrophil nor platelet recovery differed between the two arms. Furthermore, no significant difference was observed in the amount of infused suspension of RBC or apheresis platelets between DAC+IA and IA arm.

**Table 2:** Hematologic toxicity during induction treatment

Toxicity	IA (n = 37)		DAC+IA (n = 46)		*P* value
	n	%	n	%	
Granulocyte decreased (Grade 4)	37	100	46	100	
Granulocyte recovery, days					
Median	19		20		.168
Range	16‐24		8‐36		
Platelet decreased (Grade 4)	37	100	46	100	
Platelet recovery, days					
Median	18		18		.778
Range	13‐24		11‐26		
Platelet transfusions, U					
Median	5		5		.294
Range	2‐14		2‐13		
Anemia					
Grade 1‐2	0	0.0	1	2.2	.610
Grade 3	1	2.7	2	4.3	
Grade 4	36	97.3	43	93.5	
RBC transfusions, U					
Median	6		6		.796
Range	1.5‐24		0‐16		

Abbreviation: RBC, red blood cells.

Nonhematologic toxicities mainly referred to infections. Pulmonary infections had the highest incidence rate. Other common infections included upper respiratory infection (URI), oral infection, and skin soft‐tissue infection. There was no obvious difference in nonhematologic toxicities between the groups (Table 3).

**Table 3:** Most frequent nonhematologic toxicities

Toxicity	IA (n = 54)		DAC+IA (n = 54)		*P* value
	n	%	n	%	
Vomiting					
Grade 0	49	90.7	44	81.5	.334
Grade 1‐2	4	7.4	9	16.7	
Grade 3‐4	1	1.9	1	1.9	
Diarrhea					
Grade 0	53	98.1	50	92.6	.360^a^
Grade 1‐2	1	1.9	4	7.4	
Rash					
Grade 0	51	94.4	50	92.6	1.000^a^
Grade 1‐2	3	5.6	4	7.4	
ALT/AST increased					
Grade 0	50	92.6	48	88.9	.507
Grade 1‐2	4	7.4	6	11.1	
ALP increased					
Grade 0	52	96.3	54	100	.475^a^
Grade 1‐2	2	3.7	0	0	
Blood bilirubin increased					
Grade 0	51	94.4	49	90.7	.713^a^
Grade 1‐2	3	5.6	5	9.3	
Infection sites					
Upper respiratory	12	22.2	9	16.7	.466
Lung	25	46.3	30	55.6	.336
Tooth/gum/lip	14	25.9	13	24.1	.824
Skin soft tissue	10	18.5	12	22.2	.633
Anorectal	4	7.4	4	7.4	1.000^a^
Sepsis	5	9.3	5	9.3	1.000
Septic shock	1	1.9	2	3.7	1.000^a^
No infections	2	3.7	0	0	.475^a^

Abbreviations: ALP: alkaline phosphatase; ALT: alanine transaminase; AST, Aspartate aminotransferase.

a Analyzed by Fisher's exact test.

### 2.5 Survival

With a median follow‐up of 6.7 months, the 1‐year probability of OS and PFS for the whole group was 86.8% (SE ± 4.3%) and 81.9% (SE ± 5.0%), respectively. No significant difference between the study arms could be demonstrated with respect to 1‐year OS (DAC+IA 91.0% vs IA 84.2%, *P* = .991) and PFS (DAC+IA 90.6% vs IA 76.8%, *P* = .826). Long‐term survival analysis requires a longer duration to follow‐up.

## 3 DISCUSSION

According to the WHO classification criteria for AML in 2008, AML with MRCs should either include a history of MDS or have no MDS history but be associated with MLD or an MDS‐related cytogenetic abnormality, all of which suggest a poor prognosis.

Compared with 187 cases of AML‐NOS, Xiao‐Qian Xu et al^13^ reported that markedly lower WBC counts and hemoglobin were observed in AML with MLD and MDS‐related cytogenetic abnormality, 36.5% of which had a complex karyotype. It is also reported that the genes with the highest frequencies of mutation in AML‐MRC were ASXL1, TP53, RUNX1, and DNMT3A, of 21%, 28%, 12%, and 9%,^14^, ^15^ which were predicted to a poor prognosis. The CR rate achieved in patients treated with the IA/DA regimen for induction chemotherapy was 63.6%, which was significantly lower than that achieved in the AML‐NOS patients (77.5%).^13^ The 3 + 7 regimen did not achieve satisfactory results, which was the same as the results in our study.

DAC is a DNA methylated transferase (DNMT) inhibitor when used at a low dose (5‐20 mg/m^2^ per dose) and is approved for MDS and elderly AML patients. In some refractory and relapsed (R/R) AML, it can be combined with a Cytarabine/Aclamycin/Granulocyte colony‐stimulating factor (CAG) regimen.^16^ In an open‐label phase I study performed in 2011,^17^ 30 newly diagnosed AML patients (median age at 55) were included and treated with DAC at a dose of 20 mg/m^2^ per day for 3‐7 days to achieve epigenetic priming for intensive chemotherapy; this treatment was followed by daunorubicin (DNR) and Ara‐C (DA) regimen. The total response rate was 90%, with 57% (17/30) achieving CR and 33% (10/30) achieving PR. In 2015, Jiang et al^18^ analyzed the treatment response of the DAC‐sequential HAA regimen (Homoharringtonine+cytarabine +aclarubicin) in high‐risk or R/R AML patients aged 16 to 59 years old and found an overall CR rate after two cycles of up to 65.2%, which was better than the HAA group (41.7%). However, the use of DAC combined with chemotherapy‐induced therapy in newly diagnosed AML patients with MDS features under 60 years old has rarely been reported.

Considering that some AML patients show the characteristics of MDS, we suggest that DAC could be added to AML chemotherapy to improve its efficacy. The potential increase in side effects caused by this combination needs to be further explored. In our retrospective study, the CR rate after the first induction therapy was, therefore, significantly better than the historical control (85.2% vs 68.5%, *P* = .040), and there was no difference in either hematologic or nonhematologic toxicities. Multivariate analysis indicated that the combination with DAC were independent factors for CR rate after the first induction therapy.

We therefore propose that DAC plus chemotherapy is an option for initial induction treatment in AML with features of MDS, which can improve outcomes to some extent without increasing treatment‐related side effects.

Which group of patients can benefit from the combination of DAC and chemotherapy? The subgroup analysis showed that intermediate karyotype, positive FLT3‐ITD mutation, or intermediate‐poor risk stratification according to NCCN criteria may be factors that predict the CR rate.^10^, ^19^

How does DAC work in combination with chemotherapeutics? In the past, some studies conducted in vitro experiments and found that combinations including DAC improved the sensitivity of leukemia cells (HL‐60, Kasumi‐1) to conventional chemotherapy drugs, such as cytarabine, aclarubicin, and HHT, by promoting apoptosis in leukemia cells.^18^ For example, caspase‐3 and caspase‐9 were expressed at significantly higher levels, and the antiapoptotic protein Bcl‐xl was expressed at lower levels.^20^ In addition, the demethylation of DAC had definitely been observed; the expression of DNMT1, DNMT3A, DNMT3B proteins or mRNAs are lower,^20^ and the demethylation of Wnt/beta‐catenin pathway inhibitors was found to have anti‐leukemia effects.^21^ However, whether this plays an important role or is simply an accompanying effect needs to be further studied.

In summary, DAC+IA represents a new option of induction therapy for newly diagnosed AML patients with MDS features, aged <60 years old, especially in intermediate‐poor risk status.

## CONFLICT OF INTEREST

The authors declare no conflict of interest.

## Data Availability

The data that support the findings of this study are available from the corresponding author upon reasonable request.
