# Supplementary material for: A favorable inductive remission rate for decitabine combined with chemotherapy as a first course in <60‐year‐old acute myeloid leukemia patients with myelodysplasia syndrome features
Source: Cancer Med. 2019 Jul 19;8(11):5108–15. doi: 10.1002/cam4.2418 (PMC6718585; doi:10.1002/cam4.2418)
Supplement: Supplementary file 1 [file CAM4-8-5108-s001.docx]

**Supplemental** **Table S1.** Characteristics of 134 patients before propensity score matching

|  | IA (n=74) | DAC+IA (n=60) | P value |
| --- | --- | --- | --- |
| Age, years |  |  | 0.826 |
| Median | 35.0 | 35.5 |  |
| Range | 15-59 | 14-60 |  |
| History, months |  |  | 0.340 |
| Median | 1.0 | 1.0 |  |
| Range | 0.2-7.0 | 0.1-13.0 |  |
| WBC, ×10^9^/L |  |  | 0.506 |
| Median | 18.28 | 25.09 |  |
| Range | 0.89-361.7 | 0.96-232.2 |  |
| MCV, fL |  |  | 0.970 |
| Median | 100.65 | 100.40 |  |
| Range | 80.3-115.3 | 68.6-120.7 |  |
| Blasts, % |  |  | 0.717 |
| Median | 54.5 | 55.0 |  |
| Range | 21.0-94.0 | 20.5-91.0 |  |
| Gender, n(%) | | | 0.676 |
| male | 43 (58.1) | 37 (61.7) |  |
| female | 31 (41.9) | 23 (38.3) |  |
| Dysplasia, n(%) | | | 0.134 |
| 0 | 67 (90.5) | 49 (81.7) |  |
| 1 | 7 (9.5) | 11 (18.3) |  |
| Cytogenetics related to MDS, n(%) | | | 0.876 |
| 0 | 66 (89.2) | 53 (88.3) |  |
| 1 | 8 (10.8) | 7 (11.7) |  |
| NCCN prognostic stratification, n(%) | | | 0.558 |
| favour | 25 (33.8) | 18 (30.0) |  |
| intermediate | 23 (31.1) | 24 (40.0) |  |
| poor | 26 (35.1) | 18 (30.0) |  |
| Complete remission, n(%) | | | 0.025 |
| 0 | 25 (33.8) | 10 (16.7) |  |
| 1 | 49 (66.2) | 50 (83.3) |  |
